# Transient destabilization of interhemispheric functional connectivity induced by spreading depolarization

**DOI:** 10.1162/netn_a_00405

**Published:** 2024-12-10

**Authors:** Daria A. Lachinova, Maria P. Smirnova, Irina V. Pavlova, Ilya V. Sysoev, Lyudmila V. Vinogradova

**Affiliations:** Institute of Higher Nervous Activity and Neurophysiology, Russian Academy of Sciences, Moscow, Russia; Saratov State University, Saratov, Russia; Peter the Great St. Petersburg Polytechnic University, St. Petersburg, Russia

**Keywords:** Functional connectivity, Spreading depolarization, Mutual information, Transfer entropy, Electrophysiology

## Abstract

Cortical spreading depolarization (CSD), a slowly propagating wave of transient cellular depolarization, is a reliable cortical response to various brain insults (stroke, trauma, seizures) and underlying mechanism of migraine aura. Little is known about CSD effects on brain network activity. Using undirected (mutual information, MI) and directed (transfer entropy, TE) measures, we studied the dynamics of cross-hemispheric connectivity associated with the development of unilateral CSD in freely behaving rats and the involvement of inhibitory transmission in mechanisms of the coupling changes. We show that the development of CSD in the cortex of one hemisphere is followed by the transient loss of undirected functional connectivity (MI) between ipsilateral and contralateral cortical regions. The post-CSD functional disconnection of the hemispheres was accompanied by an increase in driving force from an unaffected contralateral cortex to an affected one (TE). Mild cortical disinhibition produced by pretreatment with an inhibitory receptor blocking agent (penthylenetetrazole) did not affect CSD but attenuated (MI) or eliminated (TE) the CSD-induced connectivity changes. The effects of CSD on functional connectivity in awake rodents were similar at the individual and group levels, suggesting that the described connectivity response may be a promising network biomarker of CSD occurrence in patients.

## Author Summary

Cortical spreading depolarization (CSD), a wave of transient cellular depolarization, is a universal brain response to acute insults and an underlying mechanism of migraine aura. Here, we studied the effects of a single unilateral CSD induced in awake animals on cross-hemispheric functional connectivity. CSD was followed by a reversible loss of undirected functional connectivity between the affected and unaffected cortices. The post-CSD disconnection was accompanied by an increased driving force from the unaffected cortex to the affected one. The CSD-induced changes in undirected and directed connectivity were attenuated or abolished by a pharmacological blockade of intracortical inhibition. Thus, transient unilateral disfunction rapidly activates network mechanisms of inhibitory restraint from the intact to the dysfunctional hemisphere, indicating a remarkable functional flexibility of awake brain networks.

## INTRODUCTION

Growing evidence show the high flexibility of neural networks and its important role in brain plasticity. Fast alterations of interregional functional connectivity occur across behavioral states (Benisty et al., 2024; Stitt et al., 2017) and contribute to experience-dependent plasticity and learning (Bennett et al., 2018). Abnormal brain activity, such as seizures, has been shown to produce dynamic fluctuations of functional interactions in neural networks (Kramer & Cash, 2012; Medvedeva et al., 2023; Sysoeva et al., 2016). However, little is known about the network effects of cortical spreading depolarization (CSD), a universal brain response to various brain insults (stroke, traumatic brain injury, seizures) and an underlying mechanism of migraine aura (Hadjikhani et al., 2001; Hartings et al., 2014; Leao, 1944; Somjen, 2001).

CSD is a wave of intense cellular excitation that slowly propagates over the cortex (with a velocity of 3–9 mm/min) and produces transient dysfunction of the invaded area (Leao, 1944; Somjen, 2001). CSD is accompanied by a massive release of glutamate and a short-lasting enhancement of excitatory transmission in the affected tissue. Given that the intense excitatory drive developing in a separate node of a neural network is countered by a powerful feedforward inhibition (Trevelyan, 2016), we hypothesize that CSD increases intracortical inhibition counteracting the excess of excitation and thereby contributing to network effects. To test the hypothesis, we studied the time-evolving dynamics of functional connectivity between the homotopical regions of the motor cortex of the two hemispheres induced by a single unilateral CSD in freely behaving rats. The regions are interconnected to each other, mainly through the corpus callosum, the largest white matter pathway of the brain. The strong bidirectional structural and functional connectivity are reciprocal, show a high degree of flexibility, and provide interhemispheric transfer of information for shaping sensory, motor, and cognitive functions (Innocenti et al., 2022). Numerous studies have shown predominance of inhibitory interactions across the two hemispheres mediated by the gamma-aminobutyric acid (GABA) neurotransmission (Dancause et al., 2015; Spalletti et al., 2017; Xerri et al., 2014).

Being induced in the cortex of one hemisphere, CSD develops as a unilateral event not invading the contralateral cortex. Unilateral CSD evokes reversible inactivation of cortical neurons of one hemisphere, including callosal ones. Imaging and electrophysiological studies have shown that unilateral CSD transiently reduces cross-hemispheric functional interactions (Li et al., 2012; Vinogradova et al., 2021), but the nature and potential mechanisms of the CSD effects remained unclear. In the present study, we addressed the point using the analysis of directed measure of functional connectivity and pharmacological modulation of GABAergic transmission. We computed functional and effective connectivity from bilateral local field potential (LFP) signals obtained before and after initiation of a single unilateral CSD in awake, freely behaving rats. To assess functional connectivity, we used the mutual information (MI) function, a relatively simple nonlinear measure of dynamical coupling between two signals. MI function demands a few data in comparison with Granger causality and similar techniques, being a robust nonparametric measure used in neuroscience for more than 40 years (Mars & Lopes da Silva, 1983; Novelli & Lizier, 2021; Timme & Lapish, 2018). However, it is not able to address the directionality of the connectivity changes and reveal the causal influence. Therefore, we also used transfer entropy (TE), a model-free method to detect directed interactions between time series (Schreiber, 2000) that is sometimes considered as a version of nonlinear Granger causality (Barnett et al., 2009). A close relationship between TE values and the strength of synaptic/structural connections in the same direction (Honey et al., 2007; Ursino et al., 2020) makes the measure a valuable approach to analyze directed functional interactions in neural networks and is frequently used to assess connectivity from EEG/MEG (magnetoencephalography) data. A role of GABAergic inhibitory processes in mechanisms of CSD-induced connectivity changes was evaluated in animals with pharmacologically reduced GABAergic inhibition by pretreatment with penthylenetetrazole (PTZ), an antagonist of GABA(A) receptors.

## RESULTS

### Undirected and Directed Connectivity Between the Cortices of the Two Hemispheres After Initiation of Unilateral CSD

**Figure 1.**
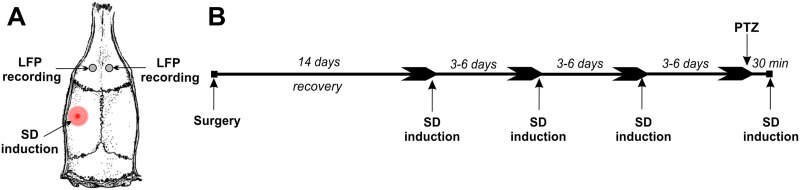
Localization of recording electrodes, site of SD induction (A) and a scheme of experimental design (B).

Figure 1 shows the experimental design of the study. A single unilateral CSD was induced by a pinprick of the left somatosensory cortex, and the LFP signals from the frontal cortex of the two hemispheres were recorded in freely behaving rats using preliminary implanted recording electrodes. CSD was induced repeatedly; in the last test, CSD was triggered after systemic administration of PTZ.

**Figure 2.**
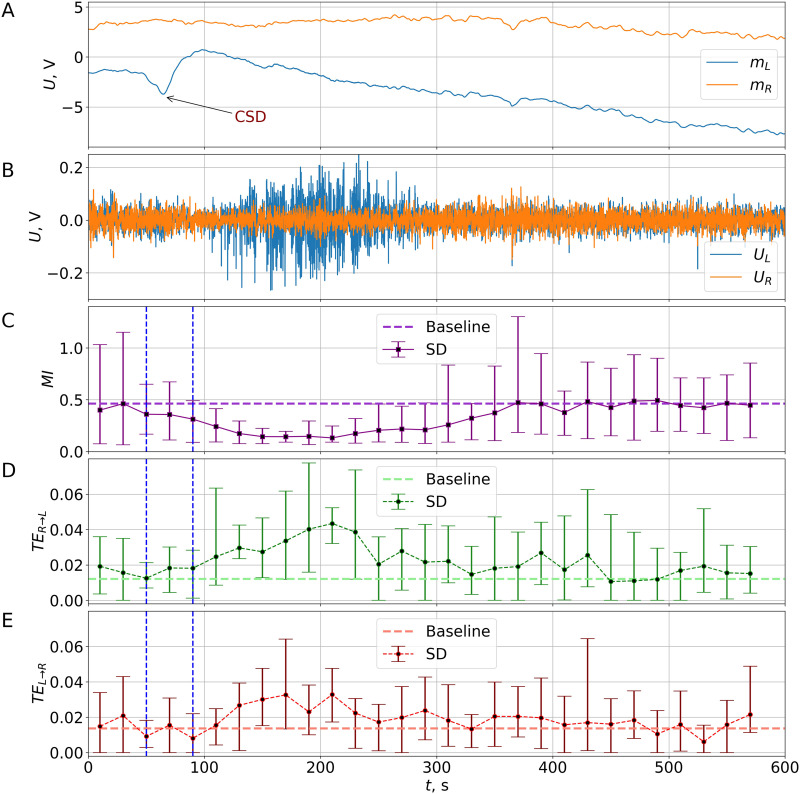
(A–B) Typical DC/AC recordings of unilateral CSD in an awake rat. Time series of low-frequency (moving average, i.e., trend) (A) and high-frequency (detrended, *f* ≥ 0.5 Hz) (B) components of LFP signals measured in the homotopic sites of the frontal cortex of the left (light blue) and right (orange) hemispheres after the initiation of CSD in the left somatosensory cortex at the time moment *t* = 0. (C) The dynamics of the MI function between frontal cortices of the two hemispheres, calculated in the nonoverlapping windows of *T* = 20-s length after the initiation of unilateral CSD. The squares show the mean values averaged over *N* = 8 rats. The error bars show the minimal and maximal (not standard) values. The mean duration of CSD (depolarization phase of the left-sided CSD) is marked by vertical dashed lines. The horizontal dashed line shows the mean value calculated from the whole baseline activity of all rats (averaging over 240 intervals of 20 s in length). (D–E) Effect of unilateral CSD on directional connectivity between frontal cortices of the two hemispheres. Values of the TE calculated in 20-s nonoverlapping time intervals in both directions: from right to left (upper subplot, the direction to the affected hemisphere) and from left to right (lower subplot, the direction from the affected hemisphere). The average, minimal, and maximal values are shown (*n* = 8). Other marks are the same as in (C).

Figure 2A shows a typical low-frequency (*f* < 0.5 Hz) component of the LFP signals obtained from the right and left frontal cortices (moving averages *m*_*L*_(*t*) and *m*_*R*_(*t*)) immediately after CSD induction in the left somatosensory cortex of a freely behaving rat. A negative slow potential shift heralded the arrival of CSD to the left frontal cortex in 46.8 ± 1.5 s (*n* = 14) after stimulation. Given the distance of 4.7 mm between the sites of CSD initiation and its registration, the velocity of CSD propagated over the cortex was of about 6 mm/min. The DC (direct current) potential shift associated with CSD lasted until 85.7 ± 1.6 s, that is, the duration of the depolarization phase of CSD consisted of about 40 s (marked by dashed lines on Figure 2C–E). The negative DC potential drift observed after CSD termination seemed to reflect slow potential changes produced by nonneuronal events (Herreras & Makarova, 2020; Voipio et al., 2003) and/or electrode polarization (low-frequency noise). Figure 2B shows a filtered high-frequency (*f* ≥ 0.5 Hz) AC (alternating current) LFP recording of the same fragment. As seen, a significant increase in ipsilateral cortical activity followed CSD. The post-CSD ipsilateral cortical hyperactivity was recorded in all rats except one (*n* = 13) during the epoch from 106.5 ± 1.9 s to 328.5 ± 39.4 s.

The dynamics of MI, the undirected measure of functional connectivity, after initiation of CSD in the left hemisphere is shown on Figure 2C. The mean value of MI did not change during the depolarization phase of CSD (marked by dashed lines) but started to reduce after CSD termination and remained decreased until 300 s. After unilateral CSD, the mean value of MI decreased during nine time intervals: from *t* ∈ [100; 120] s to *t* ∈ [260; 280] s, that is, for 180 s. After the correction for multiple comparisons, MI fell significantly below the baseline with the significance level *p*′_8*c*_ < 0.0003 at least for four double intervals: *t* ∈ [100; 140], *t* ∈ [140; 180], *t* ∈ [180; 220], and *t* ∈ [220; 260] s. Second, the dispersion and absolute difference between the maximal and minimal values of MI denoted as ΔMI became much smaller; indeed, ΔMI ≈ 0.12 for the *t* ∈ [160; 180]-s time interval, but it was in the range MI ∈ [0.9; 1.15] before CSD propagation (first two intervals).

So, MI analysis produced two main outcomes: (a) The MI between signals from the two hemispheres significantly dropped (up to fourfold) after the CSD for at least 180 s starting from 100 s; (b) the diversity between animals reduced (up to ninefold) during the same post-CSD period. Thus, for all animals, the signals in both hemispheres became very different, irrespective of their similarity before CSD (we considered MI as a quantitative measure of similarity, with its mean value being about 0.45 and its spread being ~1 just after injection). Remarkably, maximal dissimilarity (minimal average value of MI ≈ 0.13 and its spread of ~0.12) was observed not during the depolarization phase of CSD (DC potential shift) but after its termination when the amplitude of the ipsilateral AC signal increased. Interhemispheric similarity returned to the pre-CSD baseline level by 300 s simultaneously with recovering AC activity.

Next, we examined the effect of unilateral left-sided CSD on the directed measure of interhemispheric connectivity – TE that reveals causality rather than similarity between signals. Figure 2D–E shows the TE dynamics from the intact (right) cortex to the affected (left) one (D) and in the opposite direction (E). Although the baseline levels (horizontal dashed lines) of interhemispheric coupling were absolutely the same for both directions (the difference was less than 0.001), the TE dynamics following CSD differed.

There was no difference between TE_*R*→*L*_ values before and during CSD, but after CSD termination, its level changed. At least, during four 20-s intervals, the driving force from the right cortex to the left one became significantly larger than the baseline level (Figure 2D–E). For the neighbor intervals *t* ∈ [160; 180] s and *t* ∈ [220; 240] s, the minimal TE values lay very close to the average mean value. After correction for multiple comparisons following Maris and Oostenveld (2007), TE_*R*→*L*_ significantly increased compared with the baseline (*p*′_8*c*_ < 0.0003) at both double intervals: *t* ∈ [120; 160] s and *t* ∈ [200; 240] s, peaking (threefold) at 200–220 s. Thus, after termination of unilateral CSD, the unaffected hemisphere increased its influence on the affected one.

The coupling in the opposite direction did not change significantly. After applying the Bonferroni correction for double intervals, there was no double (40 s in length) intervals in which an increase in TE_*L*→*R*_ was significant at the chosen level *p*′_8*c*_ < 0.0003. Thus, unilateral CSD did not modify the influence of the CSD-affected cortex on the opposite unaffected cortex.

### Effects of PTZ on CSD-Induced Interhemispheric Connectivity Changes

**Figure 3.**
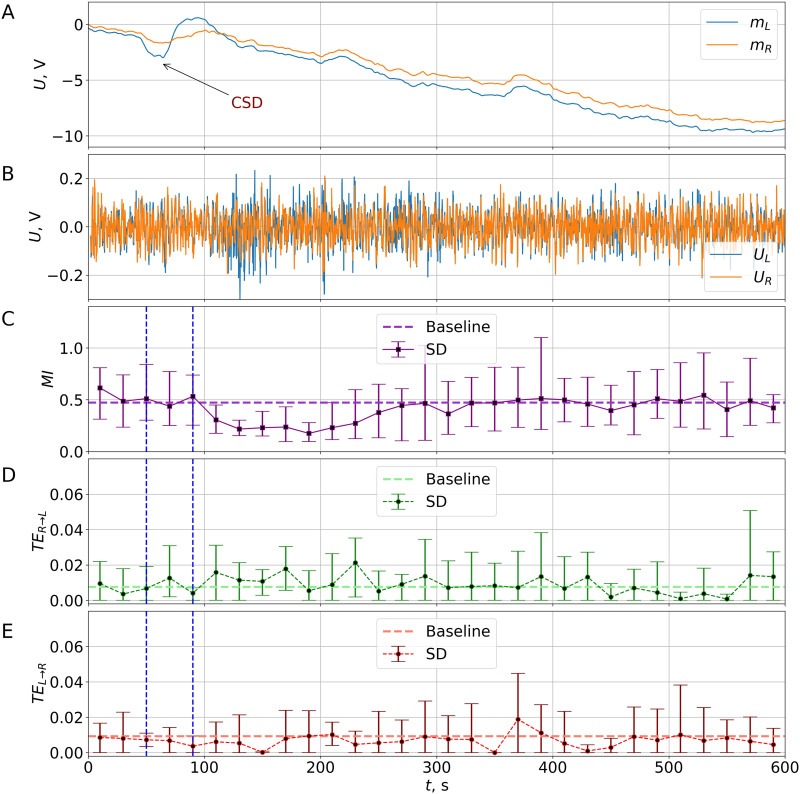
(A–B) Typical DC/AC recordings of unilateral CSD in an awake PTZ-treated rat. Time series of low-frequency (moving average, i.e., trend) (A) and high-frequency (detrended, *f* ≥ 0.5 Hz) (B) components of LFP signals measured in the homotopic sites of the frontal cortex of the left (light blue) and right (orange) hemispheres after the initiation of CSD in the left somatosensory cortex at the time moment *t* = 0. (C) The effect of unilateral CSD on undirected interhemispheric MI connectivity in PTZ-treated rats. The dynamics of the MI function, calculated in the nonoverlapping windows of *T* = 20-s length after the initiation of CSD in animals administrated with PTZ. The squares show the mean values averaged over *n* = 5 rats. The error bars show the minimal and maximal values. The time interval of the depolarization phase of CSD is marked by vertical dashed lines. The horizontal dashed line shows the mean value calculated from the whole baseline activity of all rats (averaging over 150 intervals of 20 s in length). (D–E) Effect of unilateral CSD on directed interhemispheric connectivity in PTZ-treated rats. Values of the TE calculated for animals administrated with PTZ in 20-s nonoverlapping time intervals in both directions: from right to left (upper subplot, the direction to the affected hemisphere) and from left to right (lower subplot, the direction from the affected hemisphere). The average, minimal, and maximal values are shown. The horizontal dashed line shows the mean value calculated from the whole baseline activity of all rats (averaging over 150 intervals of 20 s in length).

Pretreatment with a low dose of GABA(A) antagonist PTZ (35 mg/kg) led to the appearance of spontaneous bilaterally symmetrical spike-wave discharges (SWDs) in the cortex. Recurrent absence-like discharges were recorded in all PTZ-treated rats during the baseline period before CSD initiation. The mild epileptic activation of the cortex did not significantly change the CSD occurrence—cortical pinpricks elicited CSD in 63% (5/8) of PTZ-treated animals and in 93% (13/14) in untreated rats (*p* = 0.1167, Fisher test). A typical example of CSD recording in a PTZ-treated rat is shown on Figure 3A–B. In animals administered with PTZ, CSD was also followed by transient epileptiform activation of the ipsilateral cortex during about the same period (from 105 ± 5.5 to 304.0 ± 74 s) as in untreated rats (see above).

First, the baseline MI level in PTZ-injected animals was higher (0.4682) than that in untreated rats (0.4641, *p* < 0.00003). Second, in PTZ-treated rats, the MI changes induced by unilateral CSD (Figure 3C) were milder than those in untreated rats. The CSD-induced MI drop became shorter, with a significant decrease at only two double intervals: *t* ∈ [100; 140] s and *t* ∈ [140; 180] s compared with four double intervals in untreated animals (Figure 3C). The strength of the CSD-induced functional connection drop also became a bit milder: 2.3-fold (from 0.48 to 0.21) versus 3.6-fold (from 0.47 to 0.13) in rats with and without PTZ administration, respectively (when calculated from the average values of MI).

The CSD-induced dynamics of TE also differed in PTZ-treated and PTZ-untreated rats (Figure 3D–E). First, the baseline TE in both directions was approximately 1.5-fold lower in PTZ-treated animals compared with untreated ones—TE_R→L_: 0.0076 versus 0.0122 (*p* < 0.0003) and TE_L→R_: 0.0093 versus 0.0138 (*p* < 0.005), respectively. Second, no changes in driving force from the unaffected cortex to the affected one were detected after unilateral CSD in PTZ-injected rats although TE drive in the opposite direction became below the baseline at the double interval *t* ∈ [420; 460] s (*p*′_5*c*_ < 0.03) (Figure 3D–E).

### CSD-Induced Changes in Interhemispheric Coupling in an Individual Animal

To assess effects of unilateral CSD on interhemispheric functional interaction at the individual level, we analyzed LFP recordings obtained in a single rat in three repeated tests with 5-day intervals.

**Figure 4.**
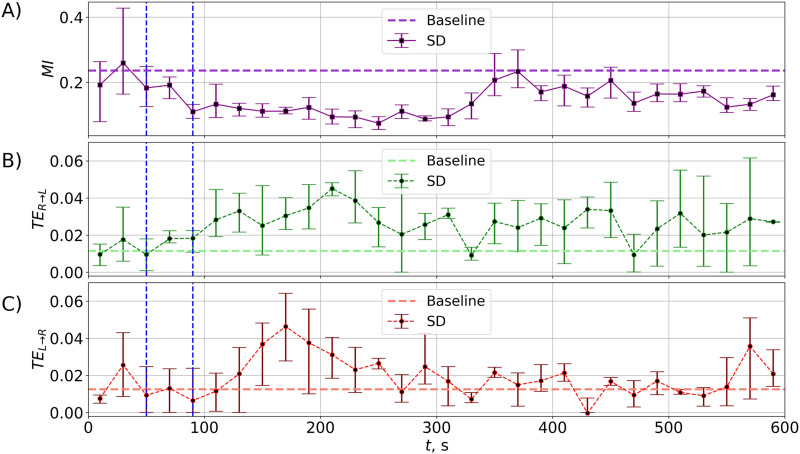
(A) MI dynamics induced by unilateral CSD in a single rat (averaged over three recordings). Abbreviations are the same as in Figures 2 and 3. (B–C) TE values for a single rat averaged over three different recordings: The upper subplot is for the direction right to left (from the nonaffected to the affected hemisphere), and the lower subplot is for the opposite direction. Error bars show minimum/maximum values.

As for the whole group, the animal showed a pronounced MI decrease after unilateral CSD that returned to the baseline level by 360 s (Figure 4). The significance level for each separate interval was insufficient (*p* = (1/2)^3^ = 0.125) due to us only having three series. Using the approach by Maris and Oostenveld (2007), considering a number of values in a row, we detected significant decreases in MI at four 60-s time intervals: *t* ∈ [60; 120], *t* ∈ [120; 180], *t* ∈ [180; 240], and *t* ∈ [240; 300]. At least for 20 s for each of these intervals, MI was smaller than the baseline at the *p* value *p*′_3*c*_ < 0.02, supporting the results obtained for MI averaged over all rats.

Considering the results of the TE calculation for this rat (Figure 4), one can find that the directed connectivity analysis showed a similar asymmetry in directed coupling as it was found for the whole population. If we took into account larger intervals (three values in a row as proposed by Maris & Oostenveld, 2007) for three tests obtained in the animal, we can find at least one interval *t* ∈ [180; 240] at which the values TE_*R*→*L*_ were larger than the baseline at the appropriate significance level *p*′*_c_* < 0.02, with no such intervals for the connectivity in the opposite direction. Thus, the TE dynamics was similar to that described at the population level when the unaffected hemisphere tended to increase its driving to the affected one. The single-animal analysis showed close similarity of MI and TE alterations in three repeated tests, indicating the reversible nature of the CSD-induced connectivity changes.

## DISCUSSION

The present study shows that unilateral CSD produces reversible destabilization of interhemispheric functional interactions and GABAergic inhibition plays an important role in the transient network crisis induced by CSD.

The analysis of the undirected measure of functional connectivity (MI) showed that unilateral CSD was followed by a transient (3-min) attenuation of interhemispheric coupling. The result is in line with previous fMRI- and EEG-based findings (Li et al., 2012; Vinogradova et al., 2021) and implies that the electrical activity of homotopical cortical regions of the two hemispheres exhibits almost an independent dynamic soon after the termination of unilateral CSD. The timing of the CSD-induced loss of interhemispheric functional connectivity coincided with post-CSD alterations of AC activity in the ipsilateral cortex. The cross-hemispheric functional connectivity remained persistent during the depolarization phase of CSD (negative DC potential shift), although it is thought that, during this phase, unilateral inactivation of callosal neurons and bihemispheric imbalance are at their maximal strength (Leao, 1944; Somjen, 2001). MI level dropped after CSD termination when ipsilateral AC activation and abnormal behavior developed (Vinogradova et al., 2021). The relationship between CSD-induced changes in functional connectivity and AC activity corresponds well to the current understanding of their basic mechanisms. Synaptic (neuronal) interactions are the main contributors to fast (AC) oscillations (Buzsáki et al., 2012) and functional coupling within neural networks (Ursino et al., 2020). The high-amplitude negative DC potential shift generated by CSD has a complex origin with contribution from multiple sources, including depolarization of neurons (synaptic and extrasynaptic membranes) and glial cells and signals from nonneuronal generators (Herreras & Makarova, 2020; Somjen, 2001; Voipio et al., 2003).

Estimation of directed connectivity, using TE measure, showed that the post-CSD disruption of interhemispheric functional similarity was accompanied by a transient (2-min) increase in driving force from the contralateral (unaffected) cortex to the affected one. That is, during the period of reduced interhemispheric similarity, the influence of the intact (healthy) cortex to the cortex affected by CSD enhanced without changes in the opposite direction. A 40-s delay between the onsets of the MI and TE changes suggests that the drive from the spared cortex may be triggered by the post-CSD loss of interhemispheric similarity of cortical activity. Experiments with PTZ showed the GABAergic nature of the post-CSD changes in cross-hemispheric functional interactions. When GABA(A) receptors were antagonized, the CSD-induced MI drop attenuated and the CSD-induced TE_R→L_ increase (enhanced driving force from the unaffected cortex) disappeared without changes in parameters of CSD per se (DC potential shift).

Interhemispheric pathways contain mainly of excitatory fibers that contact to both pyramidal and GABAergic neurons in the targeted hemisphere. Inhibitory effects providing feedforward inhibitory restraint of the contralateral side are prevalent in the corpus callosum (Innocenti et al., 2022; Xerri et al., 2014). The motor cortex of one hemisphere is known to exert inhibitory influence over the contralateral motor cortex to allow unimanual movements, and the corpus callosum is a predominant pathway through which the interhemispheric inhibition takes place (Dancause et al., 2015). After the unilateral lesion of the motor cortex, enhanced transcallosal inhibition from the intact side to the lesioned cortex has been found (Spalletti et al., 2017). Our study also shows that following unilateral CSD, the influence of the intact contralateral cortex on the ipsilateral one increases and GABAergic transhemispheric inhibition contributes to the connectivity changes or determines their development.

Unilateral CSD does not invade the contralateral cortex and does not change its blood flow and metabolism (Leao, 1944; Mayevsky & Weiss, 1991; Somjen, 2001), although it can increase fMRI-based connectivity within the unaffected cortex (Li et al., 2012) and elicit a slight suppression of spontaneous cortical activity (Medvedeva et al., 2024; Unekawa et al., 2013). It has been shown that CSD is followed by selective reduction of intracortical GABA-mediated inhibition (Krüger et al., 1996). The post-CSD disinhibition of the cortex may underlie epileptiform activation following CSD observed in our experiments and described previously in vivo and in vitro studies (Gorji & Speckmann, 2004; Vinogradova et al., 2021). It is known that epileptic activity can rapidly spread within the brain, and interneuronal networks provide powerful protective mechanisms guarding against its generalization within the brain (Trevelyan, 2016). A close relationship between the timing of post-CSD connectivity changes and epileptiform activation allows to suggest that one-sided excessive excitation produced by unilateral CSD may activate the interhemispheric inhibitory networks that oppose the transition into a prolonged hyperexcitable state and its generalization in neural networks. We speculate that the increased drive from the contralateral cortex reflects the homeostatic changes in interhemispheric functional interactions aimed at compensation of CSD-induced perturbation of ipsilateral cortical function.

An important finding of the present study is the pronounced effects of PTZ on the baseline levels of interhemispheric functional connectivity—increased resting-state level of MI and decreased levels of TE in both directions. The low dose of PTZ used in our study is known to model nonconvulsive absence epilepsy—a disorder of large brain networks involving both hemispheres. Diffuse insufficiency of GABA-ergic inhibition was supposed to underlie SWD occurrence in absent epilepsy (Bouwman et al., 2007). Increased interhemispheric connectivity was found in patients with genetic generalized epilepsy compared with healthy controls and was suggested as a biomarker of the disease (Alves et al., 2024; Bai et al., 2011; Elshahabi et al., 2015). In the current study, PTZ-treated animals exhibited spontaneous occurrence of absence-like bilaterally symmetric SWDs during the baseline period that was associated with strengthening basic similarity (MI) of cortical activity in the two hemispheres and with attenuation of their mutual influence on each other (TE), again confirming a role of inhibitory mechanisms in interhemispheric functional communication/coordination in intracortical networks.

To sum up, the study highlights remarkable functional flexibility of interhemispheric circuitry in awake rats. In the absence of chronic brain pathology, transient unilateral dysfunction (CSD) rapidly activates network mechanisms of inhibitory restraint—increased inhibitory drive from the intact contralateral cortex to the dysfunctional hemisphere. Similarly, unilateral stroke rapidly increases inhibition from the contralesional unaffected hemisphere to the lesioned one (Dancause et al., 2015). Given that CSD is a reliable component of acute cortical response to stroke, the stroke-induced CSD may contribute to the early poststroke contralesional inhibition and plasticity mechanisms.

Importantly, all the connectivity changes observed at the population level were found in individual animals. This suggests that connectivity processes accompanying CSD are mostly ergodic in a physical sense (averaging by population may be with some limitations replaced by averaging over different recording for a single animal) and that these processes manifest themselves in each (or at least in most) animal similarly. This suggests that the pattern of interhemispheric functional connectivity may be a potential biomarker of CSD. Registration of negative DC potential shift, the gold standard for CSD detection, is challenging in both humans and animals. The stereotypical pattern of interhemispheric connectivity alterations produced by repeated CSDs, which can readily be quantified from AC recordings, represents a promising approach to electrocorticography (ECoG)-based identification of CSD. To estimate the specificity and reliability of the connectivity pattern, further research in experimental models of various pathophysiological conditions is required.

Evaluation of the brain connectivity would benefit from the analysis of artificial data generated from mathematical models. In epilepsy research, investigation of connectivity in large-scale neural networks using different measures (Lüttjohann & van Luijtelaar, 2012; Meeren et al., 2002; Sysoeva et al., 2016) preceded the construction of reasonable mesoscale models and their verification based on connectivity analysis similar to that performed for experimental data (Medvedeva et al., 2020). We hope that a similar advance will be achieved in studying network effects of CSD.

## METHODS

### Animals

Male Wistar rats (3–4 months old) weighing 285–400 g (Stolbovaya Animal Breeding Center, Moscow, Russia) were used. Rats were housed in individual cages under controlled environmental conditions (a 12-hr light–dark cycle, lights on at 7:00 A.M., 20–23 °C) with free access to food and water. Experiments were carried out in accordance with the EU Directive 2010/63/EU for animal experiments, and the experimental protocol was approved by the Ethical Committee on Animal Experimentation of Institute of Higher Nervous Activity (protocol N1, 01.02.2022). Efforts were made to minimize the number of used animals and animal suffering.

### Surgery

Guide cannula for pinprick and electrodes for CSD recording were stereotactically implanted under isoflurane anesthesia 10 days before the onset of experiments. Stainless-steel guide cannulas (outside diameter 0.6 mm; inside diameter 0.4 mm) were aimed at the middle layers of the somatosensory cortex (anterior-posterior −2.8; medio-lateral 4.8; ventral 1.5) of the left hemisphere. The same length stylet was inserted into the guide cannula to prevent its obstruction. To record DC potential shifts associated with CSD, glass electrodes with an inner carbon fiber (a tip diameter of 50–100 m; Vinogradova, 2015) or nichrome electrodes were implanted in the homotopic sites of the frontal cortex (AP +1.2; ML ± 2.3, V 1.5) of the two hemispheres. The reference electrode was placed over the cerebellum. Electrodes and guide cannulas were soldered to a pin connector and were fixed on the skull with acrylic cement.

### Experimental Design

Experiments were performed in awake, freely moving rats with simultaneous video monitoring of behavior and recording of CSD-related DC potentials. Each rat was individually placed in a shielded experimental chamber (60 × 40 × 40 cm), and the implanted connector was attached to the recording cable. The electrical activity of the cortex (in a frequency band from 0 to 45 Hz) was recorded with a four-channel, high-input impedance (1 g) DC amplifier and analog-to-digital converter (E14-440, L-Card, Russia). The data were stored on a PC (1-kHz sampling rate) and analyzed offline. After a 5-min period of habituation in the chamber and a 10-min period of baseline DC-potential recording, microinjury (pinprick) of the somatosensory S1 cortex reliably inducing a single CSD wave was performed. A rat was gently handled, and the injection cannula (i.d. 0.3 mm) was inserted into the guide cannula, protruding from its tip (1 mm). As shown previously, cortical pinprick elicited a single CSD with 95% probability (Vinogradova et al., 2020).

Cortical DC potentials were registered for 10 min postinjury. Four repeated tests were performed at 3- to 6-day intertest intervals. In the last test, a low dose (35 mg/kg) of GABA(A) receptor antagonist PTZ (Sigma, USA) was injected interaperitoneally 30 min before CSD initiation.

Fourteen artifact-free LFP recordings of CSD-associated DC potential shifts were obtained in eight untreated rats. Eight recordings (one recording per animal) were selected for the connectivity analysis (*n* = 8). Estimation of PTZ effects on CSD-induced connectivity dynamics was performed in five rats out of the eight. Data about rats used in the present study is shown in Supporting Information Table S1.

### Histology

After the end of the experiments, the rats were overdosed with chloral hydrate and perfused intracardially with 0.9% saline. Brains were removed, stored in the fixate for 48 hr, sectioned in a cryostat in 35-μm coronal slices (Leica VT1200S, Germany), and stained with 0.1% cresyl violet. The slices were used to verify the cannula and recording electrodes positions and to evaluate the volume of tissue damage produced by the cannula insertion.

### Data Preprocessing

Experimental data contained two types of distortion, which had to be removed before the application of any connectivity measure. The first was the slow trend of the mean. We removed it by extracting the mean in the 2-s moving time window (window semilength *T* = 1 s); see Equation 1:

$$v_{n}^{\prime }=v_{n}-\frac{1}{\left(2m+1\right)}\sum_{i=n-m}^{n+m}v_{i},$$ (1)

where *v*_*n*_ is a measured value, *v*_*n*_′ is a corresponding detrended value, and 2*m* = ⌊*T*/Δ*t*⌋. The detrended signals were further analyzed. Second, high-amplitude artifacts were removed. Some of them were related to the end of analog-to-digital converter dynamical range, so it was only necessary to add 10 or −10 V to the signal. Recordings with more complex and long-lasting artifacts were excluded from the analysis.

#### Methods for connectivity reconstruction.

Here, we applied two measures for functional connectivity estimation. The first one is MI function, which is a very popular and relatively simple nonlinear measure of signal similarity described, for example, by Shannon and Weaver (1949) and used in neuroscience for at least 40 years (Mars & Lopes da Silva, 1983, 1987). MI demands a few data in comparison with other nonlinear measures like TE or Granger causality (see Kraskov et al., 2004, for details) if using modern techniques of estimation and was successfully applied in neuroscience in the moving window when studying other disorders like epilepsy (Sysoeva et al., 2016). It has also been recently applied to estimation of intracortical connectivity dynamics associated with CSD (Medvedeva et al., 2023). However, MI cannot reveal the connectivity direction and measures only signal similarity. To reveal the connectivity direction, we used another well-known approach—TE (Schreiber, 2000). The TE is sometimes considered as a version of nonlinear Granger causality (Barnett et al., 2009), and there are direct analytical proofs that, for some very simple signals, there are direct formulas to calculate TE from prediction improvement of Granger causality and vice versa.

To reveal the connectivity evolution, one has to calculate all measures in time intervals (windows) rather than from the whole series. The use of both overlapping and nonoverlapping intervals provides some advantages and disadvantages. Overlapping intervals are often used to increase the temporal resolution. However, in our case, the processes are rather slow. The problem of overlapping windows is that it is hard to make corrections for multiple testing since the results are interdependent, especially when the statistical distribution of estimates is unknown. Therefore, all measures were calculated in 20-s nonoverlapping time intervals for 600-s recordings of cortical activity obtained during the baseline period (30 intervals) and after the initiation of wave of spreading depression (*K* = 29 intervals). For each *i*-th (*i* = 1, 2, …, *K*) time interval for each rat and each experiment (if there were more than one experiment for this rat), three values were calculated: MI*_i_*, TE_*L*→*R,i*_, and TE_*R*→*L,i*_, where *L* → *R* means the direction from left to right and *R* → *L* means the direction from right to left.

### MI Function

MI function (Equation 2) can be considered as a generalization of correlation function, which takes into account all types of signal interdependencies: not only linear as correlation does, but also nonlinear.

$$\text{MI}\left(X,Y\right)=H\left(X,Y\right)-H\left(X|Y\right)-H\left(Y|X\right),$$ (2)

where *H*(*X*, *Y*) is the joint entropy of signals *X* and *Y* and *H*(*X*∣*Y*) and *H*(*Y*∣*X*) are conditional entropies. If the entropies were calculated by mean of estimation of probability density from bins (i.e., by calculating the probabilities, *p*_*Y*_(*j*) and *p*_*X*,*Y*_(*i*, *j*) that the corresponding value (*x*_*n*_, *y*_*n*_) belongs to *i*-th bin by *X* and *j*-th bin by *Y*), the calculation formula would be the following (Equation 3):

$$\text{MI}\left(X,Y\right)=\sum_{i,j}p\left(i,j\right)\text{log}\frac{p\left(i,j\right)}{p\left(i\right)p\left(j\right)}.$$ (3)

In the past, some nonlinear correlation coefficients were actively used in neuroscience (e.g., Pijn et al., 1989). This was mostly forced by the difficulties in calculating MI directly from bins using Equation 3 and due to troublesome properties of differential entropy (Moddemeijer, 1989). This problem was solved by Kozachenko and Leonenko (1987) by introducing a new estimate via account of the nearest neighbors instead of direct calculation by bins, but this mathematical work remained unused until the calculation algorithm was described only by Kraskov et al. (2004) in detail. This approach was tested and found to be the best one in terms of precision and computation time; see Papana and Kugiumtzis (2009). Therefore, MI should now be preferred in comparison with other nonlinear similarity measures since it is the most universal and straightforward approach. Now, to calculate MI, one should introduce the distance between data points (*x*_*i*_, *y*_*i*_) and (*x*_*j*_, *y*_*j*_) as a maximum of absolute values (Equation 4).

$$d_{i,j}=\text{max}\left(|x_{i}-x_{j}|,|y_{i}-y_{j}|\right).$$ (4)

Then, for each data point (*x*_*i*_, *y*_*i*_), the *k*-th nearest neighbor is detected using Equation 4, and the number of points that are *X*-neighbors of (*x*_*i*_, *y*_*i*_) (*n*_*X*_(*i*)) and *Y*-neighbors (*n*_*Y*_(*i*)) is calculated. So, the resulting formula is the following (Equation 5):

$$\text{MI}=\psi \left(N\right)+\psi \left(k\right)-{\left\langle \psi \left(n_{X}\left(i\right)+1\right)+\psi \left(n_{Y}\left(i\right)+1\right)\right\rangle }_{i},$$ (5)

where *N* is the number of data points and *ψ* is the digamma function. Estimates of MI ignore all continuous linear signal transformations, including signal shift and scaling.

The only method parameter is *k*. Usually, *k* > 1 is recommended. We tested our results for stability using *k* = 1, *k* = 6, and *k* = 10 and found no significant difference. Therefore, we used *k* = 1 for all cases to simplify calculations.

### TE

TE was proposed by Schreiber (2000) as an alternative realization of the same concept as the nonlinear Granger causality (Ding et al., 2006; Marinazzo et al., 2006, 2008; Sysoeva et al., 2014). It has been actively used in neuroscience for the last decade (Vicente et al., 2011). The idea is that, if the (*i* + *τ*)-th value of the series ${\left\{x\right\}}_{i=1}^{N}$ is determined not only by its previous *i*-th value but also by the previous *i*-th value of the series ${\left\{y\right\}}_{i=1}^{N}$ (so, we suggest that the object *Y* drives the object *X*), then the conditional entropy *H*(*x*_*i*+*τ*_∣*x*_*i*_, *y*_*i*_) is smaller than the other conditional entropy *H*(*x*_*i*+*τ*_∣*x*_*i*_), where *τ* is an analog of Granger causality prediction time as proposed by Sysoeva et al. (2014). The difference between these two conditional entropies is the amount of information transferred from *Y* to *X* between the time moments number *i* and *i* + *τ*. In particular, the definition is as follows:

$${\text{TE}}_{Y\to X}=H\left(x_{n+\tau }|x_{n}\right)+H\left(x_{n+\tau }|x_{n},y_{n}\right).$$ (6)

If we consider the signal to be ergodic, we can estimate the entropies by averaging over time instead of averaging over different series. This would lead us to the calculation formula similar to Equation 3. However, such an approach is very inefficient due to the necessity of estimation of probabilities in three-dimensional space. Therefore, here, we used the same nearest neighbor approach as for MI calculation, but generalized to three dimensions. This idea is more or less trivial and was simultaneously proposed by different groups; see Sysoev and Sysoeva (2015), Zhu et al. (2015), and Bossomaier et al. (2016), providing the calculation for Equation 7.

$${\text{TE}}_{Y\to X}=\psi \left(k\right)+{\langle \psi \left(n_{X}\left(i\right)+1\right)-\psi \left(n_{X,Y}\left(i\right)+1\right)-\psi \left(n_{X,X_{\tau }}\left(i\right)+1\right)\rangle }_{i},$$ (7)

where *n*_*X*_(*i*) is the same as in Equation 5, and *n*_*X*,*Y*_(*i*) and *n*_*X*,*X*_τ__(*i*) are calculated as the numbers of neighbors for both *X* and *Y* or both *X* and *X*_*τ*_ (e.g., *X* shifted in time at *τ* data points in the future) accordingly.

Calculation of TE by means of Equation 7 has two parameters: *k* and *τ*. We tested our method for *k* = 1, *k* = 6, and *k* = 10 at *τ* = 1 and found no significant difference. We also used two different values of *τ*, *τ* = 1 and *τ* = 15 (to use some main time scale in the signal as it is recommended by Sysoeva et al., 2014), and found the results mostly similar, but the absolute values for *τ* = 15 are very small. So, further, we report the results obtained for *k* = 1 and *τ* = 1.

#### Statistical evaluation of connectivity estimates.

Since both MI and TE are nonnegative by definition, if one considers the estimates obtained for a particular time interval as a sampling, they would have unknown and definitely nonnormal distribution. Therefore, we avoided using metrics based on the normal distribution assumption and used the minimum–maximum criteria.

First, we calculated the mean values for both metrics (for TE in both directions) for the baseline activity by averaging the overall time intervals for all animals (240 in total for eight untreated animals and 150 in total for the five animals administrated with PTZ). Large averaging of these values led to a very small standard of the mean (0.003 for MI and 0.001 for TE), so we neglected it—all comparisons were done with the empirical mean as it would be the expected value.

Second, we calculated the minimum and maximum values among all *L* animals (*L* = 8 for the initial experiment before PTZ injection and *L* = 5 for the experiment after PTZ injection) for each metric for each time interval. Then, we considered the zero hypothesis that these values had the same mean as the mean for the baseline. If so, the probability for a particular value that it would be larger (smaller) than the mean for the baseline was half, and for all *L* values, these probabilities were independent. This led to the decision that the probability to have all *L* values larger than the mean baseline value is (1/2)*^L^*. So, if the minimal value was larger than the baseline value, we had to refute the hypothesis with the mistake probability (usually known as *p* value) *p*_8_ = (1/2)^8^ < 0.004 for the pre-PTZ experiment and *p*_5_ = (1/2)^5^ < 0.032 for the experiment after PTZ injection. This level of significance was usually accepted. However, since we tested for coupling in 29 segments, we had to make a correction for multiple comparisons. Applying the Bonferroni correction means gave the values *p*_8*c*_ = 0.113 and *p*_5*c*_ = 0.906, which were not acceptable. Therefore, to provide more significant results, we used the approach by Maris and Oostenveld (2007), considering *M* subsequent intervals together. If, at each of them, the results were significant at the value *p*, this meant that, at least at one of them (we do not know at which actually), the results were significant at the level *p^M^*. This also meant that due to a worse temporal resolution, we now only had *K*′ = *K*/*M* independent intervals. So, for Bonferroni correction, we only needed to multiply *p* by *K*′ rather than by *K*. For both experiments (before and after PTZ injection), *M* = 2 was sufficient to provide well-acceptable levels of*p*′_8*c*_ < 0.0003 and *p*′_5*c*_ < 0.015.

Apart from analyzing both MI and TE measures for the population, we calculated them for three repeated experiments in a single animal. We had three recordings for the animal and used *M* = 3 to obtain the reasonable level of significance: *p*′_3*c*_ = ((1/2)^3^)^3^ · 10 < 0.02.

The MI and TE baseline levels before and after PTZ administration were compared. Since the same animals were used in the experiments and we had at least thirty 20-s intervals for each of them, based on the central limit theorem, we expected that the mean MI for each recording is distributed according to a law close to normal. Therefore, a one-sided *t* test for independent samplings was used; the realization was taken from the popular scipy.stats package (Virtanen et al., 2020).

Results were expressed as mean ± minimum/maximum for MI/TE and mean ± *SEM* for parameters of CSD and post-CSD cortical activation. The level of significance was set to *p* < 0.02 for all statistical tests. The incidence of CSDs in untreated and PTZ-treated animals was estimated using a Fisher exact test.

## ACKNOWLEDGMENTS

This work was supported by Russian Science Foundation, grant number 22-15-00327.

## SUPPORTING INFORMATION

Supporting information for this article is available at https://doi.org/10.1162/netn_a_00405.

## AUTHOR CONTRIBUTIONS

Daria A. Lachinova: Data curation; Formal analysis; Software; Visualization. Maria P. Smirnova: Data curation. Irina V. Pavlova: Data curation. Ilya V. Sysoev: Methodology; Software; Supervision; Validation; Writing – original draft. Lyudmila V. Vinogradova: Conceptualization; Funding acquisition; Supervision; Validation; Writing – original draft.

## FUNDING INFORMATION

Lyudmila V. Vinogradova, Russian Science Foundation (https://dx.doi.org/10.13039/501100006769), Award ID: 22-15-00327.

## Supplementary Material



## TECHNICAL TERMS

Cortical spreading depolarization: A self-propagating wave of massive cellular depolarization transiently changing the electrical activity of the cortex.
Glutamate: The principal excitatory transmitter of the brain that plays an important role in cognition and memory.
Corpus callosum: The largest fiber tract connecting the cortex of the cerebral hemispheres and providing communication between them.
GABA (gamma-aminobutyric acid): The principal inhibitory transmitter in the brain. Synaptic release of GABA rapidly reduces neuronal excitability.
GABA neurotransmission: The most important driver of inhibitory processes in brain networks.
LFP (local field potential): The electrical signal recorded in neuronal tissue that reflects the summed activity of neurons near recording site.
Spike-wave discharges (SWDs): Brief epileptic seizures that involve both hemispheres and are accompanied by behavioral arrest.
