# Supplementary material for: Transient destabilization of interhemispheric functional connectivity induced by spreading depolarization
Source: Netw Neurosci. 2024 Dec 10;8(4):1383–99. doi: 10.1162/netn_a_00405 (PMC11675007; doi:10.1162/netn_a_00405)
Supplement: Supplementary file 1 [file netn-8-4-1383-s001.pdf]

Supplementary Table 1

**Rat number**	**CSD 1**	**CSD 2**	**CSD 3**	**CSD 4 + PTZ**
281	+	+	+	+
282	+	-	+	+
283	+	n.dc	+	+
284	+	n.dc	n.dc	-
285	n.dc	n.dc	+	+
287	+	+	n.dc	-
289	n.dc	+	n.dc.	-
290	n.dc	+	+	+

"n. dc" – no recording of artefact-free DC potential; **"+"** pinprick triggered CSD**; "-"** pinprick did not trigger CSD
